# NEWS – REGIONS

**DOI:** 10.7189/jogh-07-020201

**Published:** 2017-12

## Africa

➢The number of new malaria cases in Gambia fell by 40% between 2011 and 2016, whilst the prevalence of the parasite in children aged under 5 years fell to 0.2% from 4% over the same period. Gambia could be the first country in sub–Saharan Africa to eliminate malaria, but faces a shortfall in funds for the most difficult “last mile” in reaching the goal of no new malaria cases by 2020. It has a funding gap of US$ 25 million, and the possibility of “donor fatigue”, whereby donors turn their attention elsewhere as cases drop, but the country’s new leadership, under the democratically elected President Adama Barrow, may reinvigorate the fight against malaria. In addition to the usual control measures such as antimalarial drugs, bednets and spraying, Gambia has deployed technology to tackle malaria, using GPS, mobile devices and online platforms to track the implementation of the standard control measures, aided by improved internet bandwidth in rural areas. (Reuters, 12 July 2017)

➢The 2017 South Africa Health Review paints a bleak picture of South Africa’s health sector, as it battles to deliver services and reduce costs in the face of stagnant economic growth. Health expenditure will increase by 1.1% in 2016/17, 0.8% in 2017/18, and will be cut by R142 million (US$ 10 million) in 2018/19 – a 0.1% reduction. The rand has fallen 38% against the US$ from 2012 to 2016, which had hugely increased cost pressures, whilst an additional 400 000 people each year are joining the HIV treatment programme, and many new children’s vaccines have been introduced. However, the costs of ARV drugs have been kept down thanks to the central procurement of medicines. Staff costs are the main expense – most provinces have imposed restrictions on filling vacant posts, and 300 medical specialists left between 2015 and 2016. Spending on infrastructure has also been cut, and more patients are being referred to clinics rather than hospitals, to save money. However, clinic visits have been cut by 3 million in the past 2 years – although a new medicine dispensing programme enables some patients to collect their medication from designated pick–up points such as private pharmacies and schools. And finally, the management skills essential to contain costs is in short supply in the health sector. (Daily Maverick, 23 August 2017)

➢Nurses in Kenya ended a five–month strike, which had crippled the country’s health system, especially maternity, surgical and inpatient services. Kenya’s Council of Governors agreed to pay nurses an initial uniform allowance of Sh15 000 (US$ 145) – an increase from its current Sh10 000 (US$96) level. Nurses will also receive a risk allowance of between Sh20 000 and Sh25 000 (US$193 – 242) each year, depending on their role. All disciplinary cases against striking nurses will be withdrawn, and salaries will be reinstated before 31 December, and Kenya’s regions are beginning to recruit nurses after a jobs freeze. “Over 85% of Kenyans living below the poverty line cannot afford expensive healthcare, and therefore we call off the strike” said Mr Seth Panyako, the KNUN secretary–general. (The Nation, 2 November 2017)

➢There have been 91 deaths from snake–bites within 3 weeks in Nigeria, but the Federal Government moved to refute rumours that public hospitals have ran out of antidotes to snake bites. Prof Isaac Adewole, Nigeria's Minister of Health, confirmed that the Ministry of Health still has some vials of anti–snake venom in stock, from its 2016 procurement exercise where states and other treatment centres were supplied upon request. He noted that the states (Gombe and Plateau) where the 91 deaths occurred had not compiled with the new system of procurement–upon–request; and that 5 states so far have requested anti–venom stocks. He called upon states to invest in their own procurement of anti–venom treatments, and warned that the Federal Government cannot continue to procure and distribute the venom without charge. “The Federal Government is however working on Public Private Partnership arrangements for local production of anti–snake bite venom which will make the product available, affordable and accessible,” he said. (allafrica.com, 8 November 2017)

➢Zimbabwe has made progress in reducing HIV prevalence from 27% in the 1990s to less than 15% in 2017, but those of its HIV–positive citizens living in extreme poverty face daily battles against the corruption and prejudice which limits their access to vital treatment, support and care. Mr Robert Mugabe’s resignation offers Zimbabwe a chance to revitalise its HIV strategy to ensure that no–one is left behind – by prioritising and fast–tracking actions for the poorest and most marginalised people. Zimbabwe’s Health Ministry must continue to strengthen HIV programmes that target women and girls, disabled people, the elderly, prisoners and people in remote rural areas, sex workers, people in same–sex relationships and those in extreme poverty. All these groups suffer discrimination and disadvantage, and have higher risks of preventable and premature death from HIV. The government has a moral responsibility to prioritise these groups so that development does not benefit wealthier groups initially, and therefore widening inequalities. Investing in the already–piloted Electronic Health Records, which can provide high–quality data security, storage and analysis, will be essential for a low–income country with high HIV prevalence to deal with high treatment needs. Corruption must also be tackled – Zimbabwe is one of the most corrupt nations on earth – and poorer people, especially marginalised groups living with HIV/AIDS rely on public services weakened by the misappropriation of funds. (The Conversation, 30 November 2017)

## Asia

➢Mr Taro Aso, Japan's finance minister and a lifelong smoker, recently expressed doubts about the link between smoking and lung cancer. His ministry also benefits (by US$ 18 billion a year) from tax revenues on tobacco products, and owns about 33% of Japan Tobacco, the world's fourth–largest cigarette–maker. Controversy has intensified recently as the government proposes a ban on smoking inside public buildings to protect citizens against passive smoking, which kills 15 000 people in Japan each year. A group of MPs from the country's Liberal Democratic Party are campaigning against the proposed ban, supported by tobacco farmers and the restaurant industry, who fear that banning smoking from their premises – which accounts for most passive–smoking deaths in Japan – would lead to mass business closures. Japan's health ministry believes that these fears may be overstated, as non–smokers appreciate clean air (following successful health campaigns, 18% of Japan’s adults smoke). Another contradictory aspect of Japan's smoking legislation is that some cities ban public smoking on streets, whilst only encouraging smoking to be banned indoors. This often leads to smokers moving indoors to smoke, rather than smoking outside. (The Economist, 22 June 2017)

➢To date, Sri Lanka’s Ministry of Health recorded at least 300 deaths and more than 100 000 infections caused by a major dengue outbreak. Heavy monsoon rains, rain–soaked garbage, standing pools of water and poor sanitation all provide ideal living conditions for the mosquitoes which transmit the virus. The International Federation of Red Cross and Red Crescent Societies (IFRC) is expanding its emergency assistance to Sri Lanka with the Sri Lanka Red Cross in order to suppress the spread of the disease. IFRC stated that the hospitals are stretched to the limit, especially in the Western Province. According to the WHO, dengue, endemic in 100 countries with an estimated 390 million cases of infection annually, is ranked as one of the world’s fastest growing diseases. Dengue is recognisable by its flu–like symptoms that can lead to the deadly hemorrhagic dengue fever. There appears to be no end in sight for dengue danger as “the virus currently spreading has evolved and people lack the immunity to fight off the new strain,” says Novil Wijesekara, of the Sri Lanka Red Cross. *(*Reuters, 24 July 2017)

➢A group of anonymous civil society organisations, people living with HIV, and others, have written to the Global Fund to express "grave concern" over its funding to combat HIV in Cambodia, as well as the allocation process. The Global Fund is the main donor in the country’s efforts against HIV/AIDS, TB and malaria. The group expressed concern that civil society representatives have had little input into decision–making, and that participation in Cambodia’s Community Co–ordinating Mechanism (CCM) – a national multi–sectoral committee in charge of facilitating Global Fund activities in the country – is skewed towards international representatives. As a result, the civil society organisations wish to step down from the CCM. These organisations are also concerned that funding is being targeted towards international NGOs, rather than local organisations working with key affected populations. In response, the Global Fund confirmed that it is planning a consultation with these groups to discuss their concerns in a transparent manner. It also noted that out the Global Fund’s US$ 41.6 million allocation to Cambodia for 2018–20, 27% is set aside for NGO–led activities. (Phnom Penh Post, 30 October 2017)

➢Winter storms are building over northern Afghanistan, bringing snow to the mountains, thunderstorms to Kabul, rain to Lahore and Islamabad in Pakistan – and helping to clear the smog hanging over the region. Pakistan has experienced traffic accidents – with 10 people killed and 25 injured in 2 weeks – linked to poor visibility, and it also causes respiratory problems. Mr Mohammad Riaz, a Pakistani meteorologist, said that the smog is caused by dust, pollution, the burning of crops, factory and brick kiln emissions, and is expected to linger until mid–November. He advised people to wear face masks to protect themselves from respiratory ailments. Average air pollution in Pakistan’s main cities is 4 times higher than World Health Organization limits, and although overall air quality in 2017 is better than 2016, pollution levels in Lahore recently reached 18 times the healthy limit. Inevitably, as the winter weather settles, an increase in pollutants will again thicken smog to “very unhealthy” or “hazardous” levels – with human activity being the main contributor. (Al Jazeera, 16 November 2017)

➢North Korea has banned most forms of birth control, and as a result condoms are increasingly in demand as a gift item brought back by business travellers returning from China, according to sources in North Korea. Condoms are prohibited for manufacture or sale in the country, and are blocked from entry at customs posts, and they are officially considered “indecent items”. North Korea’s leader, Kim Jong Un has strongly encouraged a high birth–rate. However, the high costs of education and raising a child means that most couples limit themselves to one child, although medical professionals are banned from offering birth control and abortions, in order to raise the country’s falling birth rate. Moreover, despite being banned, sex work is widespread in North Korea, making the easy availability of condoms even more essential. (Radio Free Asia, 20 November 2017)

## Australia and Western Pacific

➢Throughout the developing world, cervical cancer is the main cancer affecting women – in 2017, an estimated 266 000 women world–wide will die from it, and 85% of them will be in developing countries. Rates of cervical cancer in the Pacific Islands are also concerning, with Melanesia being particularly at risk, with an incidence rate of 33.3/100 000 women and a mortality rate of 20.7/100 000 women. Cervical cancer can be prevented, but a systematic review of cervical cancer in the region found that its preventative programmes are inadequate despite the region’s high incidence rates. It would cost an estimated US$ 2.1 million to vaccinate all 13–year old girls against HPV, which is highly affordable, particularly if obtained through a regional bulk purchase underpinned by collective bargaining. The region has an opportunity to prioritise cervical cancer through the Framework for Pacific Regionalism, which could boost the number of countries offering HPV vacation from 60 to 80%. Improved and integrated sexual and reproductive health services would also boost cervical cancer survival rates, but political will and increased resources are essential. (Radio New Zealand, 21 July 2017)

➢According to UNAIDS, Papua New Guinea saw a 4% increase in new HIV infections between 2014 and 2016, with an estimated 2800 new infections in 2016. This follows on from a 41% decline in HIV infections from 2001 to 2009. With prevention efforts stalling, Papua New Guinea needs to reinvigorate its response but it faces a number of obstacles in ending the HIV epidemic as a public health threat by 2030. These obstacles including criminalising sex work and consensual same–sex sexual activity, high rates of gender–based violence, and the challenges of extending health services to the country's rapidly–growing young population. (Radio NZ, 25 July 2017)

➢Australia’s federal government spends US$ 4.6 billion each year on private health insurance subsidies, and the consumer group CHOICE has called on it to stop providing tax breaks and rebates for “junk” health insurance policies. These policies do not cover treatments for most illnesses, including heart attacks, stroke and cancer, or only allow the policy–holder to be treated as a private patient in a public hospital. According to CHOICE, junk policies account for 13% of all hospital and combined policies on the insurance market, but cover less than 1% of hospital treatments and services. Generally, they cover a very small number of procedures, including accidents, wisdom teeth and appendix surgery, but exclude all others. Consumes are attracted to them because of the tax breaks on offer, or without realising that they bought a dud policy with little value, and many end up with “bill shock” when they discover that their treatment is not covered by the policy. “These policies are not only poor value for consumers, but are poor value for the Australian community, who subsidise junk policies than do not reduce the strain on the public healthcare system,” CHOICE argues. They also called for simplified health insurance policies, after its survey found that 44% of policyholders found it difficult to compare policies. (Sydney Morning Herald, 4 August 2017)

➢Nationally, an unusually higher number of mumps cases was reported across New Zealand, and the country’s Ministry of Health warned of “an increased risk of further outbreaks.” The University of Otago is offering its students free measles, mumps and rubella booster shots to students who are unsure of their vaccination history, or who have not had two MMR vaccinations since 1990, following an outbreak of 10 cases. Auckland reported 51 cases in the past month – mainly amongst young people, and several were recorded in Waikato and two in Nelson. Auckland’s Regional Public Health Service has expressed concern over immunisation levels. Although most people recover from mumps, it can have serious complications, including inflammation of the tissue surrounding the brain (meningitis), inflamed testicles or ovaries, and deafness. (stuff.co.nz, 17 August 2017)

➢According to the 2016 Global Burden of Disease, 1–in–5 Australians has a mental illness or a substance abuse disorder – and despite having one of the highest life expectancies in the world, and being a world leader in treating heart disease, stroke and cancer, on the whole Australia is failing to improve its mental health. The burden of mental illness has improved little over the past 20–30 years, and the findings of the Global Burden of Disease study comes one day after the Royal Australian College of General Practitioners announced that mental health is the main condition dealt with by general practitioners. Moreover, although life expectancy is increasing in Australia, the average man will live the 10 years of his life in ill–health, and the average woman will live the last 12 years in ill–health. Mental illnesses, eg, depression, anxiety, bipolar, posttraumatic stress disorder and substance abuse account for nearly 25% of the years spent in ill–health by Australians. According to Laureate Professor Alan Lopez of Melbourne University and one of the study’s co–authors, this is partly due to the difficulties in treating mental illness. “There’s no easy fix for mental health issues. Their epidemiology, the age at which they begin and just the fundamental nature of mental illness means that it is an intensive care, long treatment process. It doesn’t mean that we don’t have treatments, it just means that they take longer. And people don’t always go for treatment as well – often they just live with their disability,” he says. (Huffington Post Australia, 17 September 2017)

## China

➢At the Chinese Communist Party’s 18th Congress in 2012, China’s government pledged to eliminate rural poverty by 2020, and to pull at least 10 million people from rural poverty each year, from 2016 onwards. Based on China’s official poverty line of 2300 renminbi (US$ 333) per year, the number of rural poor fell from 555 million in 1995 to 56 million in 2015. However, despite the falling number, China still faces a smaller, yet tougher, poverty problem. China’s social assistance programme, *dibao,* is a minimum living standard programme that targets poor households below a certain income, but despite being a national programme, it is implemented locally. A World Bank report from 2015 found that 90% of rural individuals with annual income below the threshold level did not receive *diabo* from 2007–09, echoing a report from Beijing Normal University which found that over 80% of eligible households did not receive *diabo* from 2010–11. However, poverty alleviation measures such as *diabo* are ultimately only emergency mechanisms. To truly tackle China’s poverty, the government must combine reforming *diabo* with more tailored poverty relief measures on a case–by–case basis. There is evidence that this is under way in some places, with local governments targeting poor individuals with health problems; but in the long–term, the idea that jump–starting economic growth in less–developed areas is sufficient to end poverty must be abandoned. (East Asia Forum, 26 May 2017)

➢According to the 2017 Future Health Index from Philips, China has the lowest concentration of skilled health professionals (31.5 per 10 000 people) out of the 19 countries surveyed, combined with a high risk of crippling health care costs for surgical procedures. This situation has further deteriorated over the years, worsened by the country's focus on disease–curing rather than prevention, hospital overcrowding and lack of access. The country's government is aiming to improve access and affordability for its 1.3 billion citizens, moving from disease–centred care to “big health”, to deliver a full range of health services covering the entire care continuum, with an emphasis on health and chronic disease management. One of the main areas of reform is the decentralisation of China’s multi–tiered health care system to streamline resources and improve effectiveness, and – crucially – all hospitals will share resources, expertise and information to ensure cost savings. China is also embracing big data to enable precise diagnosis and personalised health care – currently, too few data are collected or organised in a way that can be analysed. The government is focusing on accelerating the roll–out of the disease–based standard clinical data repository across a range of areas, and by 2020 three digital national databases will be established, incorporating health information, health profiles and medical records. (China Daily, 27 June 2017)

➢In an interview with *The Diplomat*, Peter Fuhrman of China First Capital Ltd, talked about the looming health challenge of Alzheimer Disease in China. He recognises that the treatment of chronic diseases, with Alzheimer at the forefront, is the largest single challenge to the country’s health system. China has made massive progress in the transformation from a rudimentary health system to expanding health coverage to all areas of the country and the vast majority of its citizens. However, this has in part led to increasing life expectancy, which has reached Western levels, and now China faces the strain of millions of older people suffering from conditions without any real treatment options. Mr Furhman calls for China to foster the development of quality treatment centres for Alzheimer patients, to lengthen and enrich their lives. This will require investments in buildings as well as specialist staff. Finally, he highlights how China is set to have 50% of the total number of people with Alzheimer Disease by 2045, and has less than 500 specialist treatment beds – overall, chronic care offers excellent investment opportunities for overseas companies. (The Diplomat, 26 September 2017)

➢China and Canada are collaborating on a US$ 1.69 million project to improve mental health treatment through the use of smartphone apps, text messaging and electronic medical records. The project, EMBED, aims to close the mental health care gap, as both countries struggle with shortages of mental health care professionals – especially in remote areas, and overburdened staff. There is potential for the app to target younger people who feel comfortable on digital platforms, and it could eg, raise an alert if a student withdraws from a class or social activities. Other apps could help someone through a crisis or depressive episode, when the individual may not wish direct contact with a mental health professional. There is much debate on the impact and potential dangers than smartphones may have on mental health, but with positive use they could help those with mental health needs. “Achieving economic health takes a comprehensive approach, and it is innovation like the Digital Hub [the project host] that will help develop new approaches to improving mental wellness on an unprecedented global scale,” said Canada’s International Trade Minister, François–Philippe Champagne. (CBC.ca, 9 November 2017)

➢Rising incomes and an underfunded – albeit universal – health system are driving urban residents towards private health care insurance. According to a recent survey by Financial Times Confidential Research, 21.7% of people have some private medical cover in addition to the state programmes. Coverage is highest amongst high–income householders – 42.5% – and amongst residents of first–tier cities, where 25.8% of residents have private insurance, compared to 17.4% in third–tier cities. Insurers across China have reported brisk sales of health policies in 2017, despite restrictions on the sales of investment–linked policies. Compared to the USA, where just 16.2% of people buy their health care insurance directly, 43.5% of people in China who buy insurance buy it directly. Indeed, China’s employers already contribute a percentage of employee salaries to the state programme and are therefore normally reluctant to pick up the costs of additional cover. However, more companies are expected to offer private health care to win or retain talent, although it is expected that self–financed premiums will remain the biggest driver of growth in this market. (Nikkei Asian Review, 14 November 2017)

## Europe

➢More than 7200 people in Romania have contracted measles since late 2016, and 30 people – mostly children – have died. This outbreak has affected several countries in Europe, with the largest outbreaks in Romania and Italy, but France, Germany, Poland, Switzerland and Ukraine are also affected. The World Health Organization (WHO) recommends two doses of vaccinations, covering 95% of the population, to ensure immunity and prevent outbreaks. However, in Romania only 80% of children receive the first dose, and 50% the second dose, due to poverty, a shortage of vaccines and poor access to health care. In response, the Romanian government is pushing through legislation that would make vaccination obligatory for children to attend school. The problem is compounded by a growing number of parents who refuse to have their children vaccinated, and some religious groups and public figures launching anti–vaccination campaigns. (Medical Xpress, 23 June 2017)

➢Singapore faces growing number of professionals using drugs, and with 66% of new substances users under the age of 30 years, its Home Affairs and Law Minister has warned of a new generation of drug users. Singapore is looking to other countries, including Iceland and Finland, for models on tackling the problem. Iceland has experienced falling numbers of young people using alcohol, cigarettes and cannabis, eg, in 1998 42% of people aged 15 or 16 years of age said that they had been recently drunk – compared to 5% in 2016, and the country now has the lowest substance use by teenagers in Europe. Iceland’s success is evidence–based, and includes connecting with young people, health education, involving parents in their children’s lives and focusing on preventive factors that decrease young people’s changes of substance misuse, plus reducing risk factors that lead them to it. Eg, parents are urged to spend more time with their children, and to ensure that they are home early. Finland has local drug prevention and outreach efforts, and all areas offer free activities, such as sports, for young people. Other young people are encouraging to act as “peer educators” to their fellows on the dangers of drugs, and like Iceland, Finland has also seen declining drug use amongst its young people. (Strait Times, 27 June 2017)

➢Russia faces severe problems with the distribution of HIV/AIDS medication, leading to many patients to rely on each other to access treatment, in an echo of the film, *The Dallas Buyers’ Club,* which was set in 1980s USA. Clinics frequently run out of medication supplies in the middle of the year, leaving patients with life–threatening gaps in their treatment – so patients began to organise and distribute drugs themselves. Eg, patients whose treatment regime had changed would send their old medication for redistribution, or – tragically – through patients who had died. Drug companies have informally provided medication, via sympathetic workers and personal contacts. Russia is almost unique amongst developed countries in having increasing numbers of new HIV infections and AIDS–related deaths. Campaigners estimate that deaths will top 20 000 in 2017, up from 18 577 in 2016, although it is impossible to say how many deaths result from erratic medication supplies. Alesei Yaskovich, part of the Aptechka network for redistributing HIV medication, hopes to create a single online resource where all pharmacy managers could post real–time information about their drugs supplies. Russia’s shortages of HIV medication is partly a result of Western sanctions, but the country’s pharmaceutical companies are not filling the gap. (Radio Free Europe, 8 October 2017)

➢According to Anna Sarzynska, owner of the Anna Dental Clinic in Gdansk, Poland, around 80% of her patients come from aboard, particularly Scandinavia but also the UK and Ireland. This is part of a trend of rising medical tourism in central and eastern Europe, which has been growing by 12%–15% each year – and in 2016, 488 000 people came to Poland for treatment. To date, the region has specialised in simple treatments that do not require prior consultation, and patients are attracted by prices that may be 2–3 times lower than at home. The growth in medical tourism is bolstered by a 2013 EU directive, which enables patients to obtain treatment in any member state and have it refunded if it is covered by their own national health care schemes. The region is also attracting patients from former Soviet republics, who often lack easy access to medical services and equipment. Mr Artur Gosk, head of the Polish Association of Medical Tourism, calls for more government support for medical tourism, and others point to Turkey – a popular destination for less complicated procedures, enhanced by government efforts, such as discounts for patients flying with Turkish Airlines and plans to introduce tax–free health care zones. However, the growth in medical tourism is not necessarily leading to improvements in public services, especially in hospitals – illustrated by Poland spending 6.4% of its GDP on health care – one of the lowest levels in the EU. (Financial Times, 20 October 2017)

➢Research from MacMillan Cancer Support and Public Health England shows that 17 000 people have survived for several years after diagnosis with 10 types of stage 4 cancer. The results of this research was revealed at the 2017 National Cancer Research Institute Conference in Liverpool. It is based upon data from England's public health registry, capturing data on people who were diagnosed with one of 10 common types of cancer between 2012 and 2013, and were still alive at the end of 2015. MacMillan Cancer Support said that these figures demonstrated the changing nature of cancer, and that patients whose prospects were previously limited could see their cancers become more “treatable” and manageable, like other chronic diseases. Other studies have suggested that cancer survival rates in the UK lag behind other European countries, and experts call for earlier diagnosis and improved access to treatments. Dr Jem Rashbass, cancer lead at Public Health England, described the registry data as “an invaluable resource in helping us to track improvements in cancer outcomes and gain more understanding of the implications for those living with and beyond a cancer diagnosis.” (The Guardian, 8 November 2017)

## India

➢India comprises 20% of the world’s population, yet the DNA sequences of its people makes up just 0.2% of global genetic databases – indeed, 81% of global genetic information is collected from people with European ancestry. According to Sumit Jamuar, the chief executive of Global Gene Corp, the shortfall in mapping global genetic diversity is an error that his company plans to rectify. Global Gene Corp aims to capture anonymized genetic data from populations and share it with academic and pharmaceutical industry researchers. It will begin by focusing on South Asia, primarily India in the first instance. Although there are issues of data storage and security with the expansion of genetic mapping – a single human genome contains 3 gigabytes of data – a better understanding of the impact of genetic variations on the function of potential drugs, or identifying population–specific targets – could cut the costs of drug development. Moreover, providing more tailored health care to the diverse and growing human population could potentially save millions of lives. “This is the future. Just imagine if we can change the health outcome for every individual – that is a phenomenal promise,” said Mr Jamuar. (BBC, 22 June 2017)

➢At least 160 people have died, and millions more displaced after heavy monsoon rains caused landslides and flooding across northern India, southern Nepal and Bangladesh. In Sierra Leone, at least 200 people died in flooding in Freetown, the country’s capital. Many of the victims in South Asia had drowned, or were trapped in collapsed houses or underneath toppled trees. According to the aid agency, Heifer International, the heavy rains hit at a particularly bad time for food supplies, just after the planting of rice crops, and large numbers of livestock were swept away. Landslides and flooding are common in south Asia during the summer monsoon season, and widespread deforestation and poor urban planning make it harder for the land to absorb rainfall, worsening the flooding. In India, flood–waters have damaged bridges, power lines and washed away thousands of homes. This has affected at least 2.5 million people, of whom 200 000 are staying in 440 relief camps. In the remote region of Assam, railway lines are flooded, so helicopters are dropping food supplies and water packets to the worst–affected areas. Further westward, in the state of Hamchal Pradesh, soldiers recovered the bodies of 46 people who had been travelling in buses which were buried by a massive landslide. India’s wildlife, national parks and endangered species have also been affected by the floods. (Irish Times, 14 August 2017)

➢The World Bank estimates that India will become a high–middle income country within the next 30 years, spurred on by economic and tax reforms. The World Bank’s Chief Executive Officer, Kristalina Georgieva, praised these reforms, saying that they have had a visible impact on foreign direct investment – which has doubled from US$ 36 billion in 2013–14 to US$ 60 billion. India has also moved sharply up the World Bank’s global Ease of Doing Business ranking. Moreover, investment in infrastructure is also fostering economic growth. Over recent years, the country has moved more than 60 million people out of extreme poverty, and has set a target of eliminating extreme poverty by 2026. Mrs Georgieva believes that India could meet this goal by 2022 – well ahead of its target. “What we have seen is remarkable overall success story of India. Extraordinary achievements in the last three decades, the per capita income has quadrupled. It was done with an eye on lifting out people out of poverty,” she said. (Economic Times, 4 November 2017)

➢A recent study found that out of the 56% of households covered by India’s government–funded health care insurance, 66% of these households who sought treatment in public hospitals, and 95% of those who sought treatment in private hospitals, had to pay for treatment. The health care insurance scheme was introduced by the Modi government in 2017, in order to scale–up and strengthen health care insurance, and to purchase services from public, not–for–profit and the private sector. 360 million people (30% of the population) in India are have health care insurance, with government–funded insurance covering 200 million people, with private insurance covering the remainder. One of the main objectives of the expansion of government health insurance was to reduce the number of households facing “catastrophic expenditure” for health care, as an estimated 60 million people each year fall below the poverty line due to health care expenses. However, the results of this study suggest that hospitals are overcharging for their services, coupled with low awareness of entitlement, means that insurance cover often only leads to a discount of expenditure, rather than cashless care. (The Telegraph, 23 November 2017)

➢Figures from the World Health Organization (WHO) and published in the World Malaria Report show that 6% of the world’s new cases of malaria, and 7% of malaria–related deaths, occur each year in India. Moreover, it accounts for 90% of cases in Southeast Asia, and the highest number of deaths in the region. According to the report, 85% of estimated vivax malaria cases occurred in just five countries (Afghanistan, Ethiopia, India, Indonesia and Pakistan) – with India having the highest share of cases at 51%, followed by 12% in Pakistan and 10% in Ethiopia. The report also criticised the weakness of India’s surveillance system – a significant contribution to India’s malaria burden. However, Union Health Minister JP Nadda is optimistic about India’s progress in malaria control, tweeting that “India has successfully reduced its new malaria cases by one–third and crossed the malaria mortality targets of 2020.” (Hindustan Times, 29 November 2017)

## The Americas

➢Venezuela's HIV treatment programme was once a model for the developing world, with free, public treatment available since 1999. It imported affordable generic drugs from India, challenged the patent monopolies of Western pharmaceutical companies, and targeted marginalised communities with the distribution of free condoms. However, the country’s political and economic crisis has left its once–leading programme in ruins. Hospitals lack the basic drugs to treat infections arising from shortages of antiretroviral drugs, and people living with untreated HIV are developing drug–resistant strains of the virus. Condoms are only available at hugely–expensive private pharmacies, and there is no infant formula milk to give to babies with HIV–positive mothers, to avoid transmission via breast milk. There are no accurate figures for HIV infections – the most reliable suggests that 200 000 people may be infected. However, due to the lack of treatment, people are dying from AIDS at rates reminiscent of the epidemic's early days in the 1980s. The only blood screening in the public health system is at blood banks, and pregnant women are not screened for HIV, so the risk of mother–to–child transmission is high. In 2016, a coalition of Venezuelan people living with HIV asked the Global Fund for help; it was refused because Venezuela is a high–income non OECD country (indeed, it has the world's largest oil reserves, and continues to export petroleum). The government, which denies there is a crisis, may not support their efforts, and there is currently no other organised campaign to bring drugs into the country. (Globe and Mail, 21 June 2017)

➢For the first time in US history, the US Food and Drug Administration (FDA) is planning to introduce new regulations on tobacco products as well as on tobacco vaporisers (eg, e–cigarettes). According to the FDA, the policy’s aim is to reduce the addictiveness of cigarettes caused by nicotine amongst US smokers, along with the number of consumers, which would eventually lead to a significantly lower amount of tobacco–related deaths and diseases. After the announcement of the new regulatory plan, shares of tobacco companies plunged dramatically – Altria Group by 17%, British American Tobacco’s by 11% while Philip Morris International Inc. dropped by 7%. Moreover, the FDA considers tobacco to be the leading cause of preventable death in the US – killing more than 480 000 people each year, and costing US$ 300 billion in health care. The FDA is now turning its attention to flavoured tobacco products, such as menthol. Tobacco companies, such as British American Tobacco (BAT) and Reynolds American Inc, are convinced that “future success will require transformative, innovative products and changing the conversation about tobacco harm reduction.” (BBC, 28 July 2017)

➢In 2016, more than 60 000 people died from drug overdoses in the USA – higher than gun homicides and road fatalities combined. In October, President Trump declared opioid addiction a public health emergency, opening additional federal assistance for treatment and pledging a crackdown on drug traffickers. Mr Trump’s response also included a call for doctors to be educated on the prescription of opiods, and stated that he will urge the Chinese President, Mr Xin Jinping, to take action over China’s production of fentanyl, a drug that is infiltrating the USA’s heroin supply and exacerbating fatal overdoses. Today’s health emergency originated when many people became addicted to prescription opioids over the past 20 years, and switched to heroin when prescription drugs ran out. A public health emergency declaration lasts for 90 days, but can be extended. The government will use the Public Health Service Act to combat the emergency, but there is a funding shortfall and additional funding must be negotiated with lawmakers. (Washington Times, 26 October 2017)

➢According to the 2017 School Weight and Height survey, conducted by the Costa Rican Ministries of Health and Education, 34% of the country’s school–age children are overweight, a large increase over previous years. Health officials highlight “drastic changes” in children’s nutrition as the driving force behind the change, and longer commutes, busier parents, lack of recreational facilities combined with easy access to junk food also makes life more sedentary. Costa Rica is not alone in the increasing prevalence of obesity, as worldwide an estimated 4.3% of boys and 6.3% of girls were overweight, and in 2015 this had increased to 30.3% of boys and 33.1% of girls. The Costa Rican Social Security Agency is bolstering its health promotion activities, and is launching heart–health programmes and improving heart monitoring services. (Costa Rica Star, 14 November 2017)

➢Child marriage was banned in Mexico in 2014, but the country’s rates of child marriage have not fallen, despite the ban and falling rates globally. According to UN data, 25% of Mexican women aged 50–54 years says they married as children, compared to 21% of women aged 20–24 years – a small change over a generation. This data also shows that 6.8 million women in Mexico married before the age of 18, and 25% of Mexican women marry under–age. In the region of Coatecas Altas, some women report that the average age of marriage is 14 years. UN Women state that marrying before 18 means that young women are more likely to be poor, have a lower education level, less job opportunities and be victims of domestic violence. There are many drivers behind Mexico’s rates of child marriage, but underpinning them is society’s perceptions of women, and women’s roles. (NPR Goats and Soda, 23 November 2017)

